# Directional and inter‐acquisition variability in diffusion‐weighted imaging and editing for restricted diffusion

**DOI:** 10.1002/mrm.29385

**Published:** 2022-07-21

**Authors:** Batuhan Gundogdu, Jay M. Pittman, Aritrick Chatterjee, Teodora Szasz, Grace Lee, Mihai Giurcanu, Milica Medved, Roger Engelmann, Xiaodong Guo, Ambereen Yousuf, Tatjana Antic, Ajit Devaraj, Xiaobing Fan, Aytekin Oto, Gregory S. Karczmar

**Affiliations:** ^1^ Department of Radiology University of Chicago Chicago Illinois USA; ^2^ Research Computing Center University of Chicago Chicago Illinois USA; ^3^ Department of Public Health Sciences University of Chicago Illinois USA; ^4^ Department of Pathology University of Chicago Chicago Illinois USA; ^5^ Philips Research North America Cambridge Massachusetts USA

**Keywords:** ADC, diffusion‐weighted imaging, inter‐acquisition variation, molecular motion, restricted diffusion

## Abstract

**Purpose:**

To evaluate and quantify inter‐directional and inter‐acquisition variation in diffusion‐weighted imaging (DWI) and emphasize signals that report restricted diffusion to enhance cancer conspicuity, while reducing the effects of local microscopic motion and magnetic field fluctuations.

**Methods:**

Ten patients with biopsy‐proven prostate cancer were studied under an Institutional Review Board‐approved protocol. Individual acquisitions of DWI signal intensities were reconstructed to calculate inter‐acquisition distributions and their statistics, which were compared for healthy versus cancer tissue. A method was proposed to detect and filter the acquisitions affected by motion‐induced signal loss. First, signals that reflect restricted diffusion were separated from the acquisitions that suffer from signal loss, likely due to microscopic motion, by imposing a cutoff value. Furthermore, corrected apparent diffusion coefficient maps were calculated by employing a weighted sum of the multiple acquisitions, instead of conventional averaging. These weights were calculated by applying a soft‐max function to the set of acquisitions per‐voxel, making the analysis immune to acquisitions with significant signal loss, even if the number of such acquisitions is high.

**Results:**

Inter‐acquisition variation is much larger than the Rician noise variance, local spatial variations, and the estimates of diffusion anisotropy based on the current data, as well as the published values of anisotropy. The proposed method increases the contrast for cancers and yields a sensitivity of 98.8% with a false positive rate of 3.9%.

**Conclusion:**

Motion‐induced signal loss makes conventional signal‐averaging suboptimal and can obscure signals from areas with restricted diffusion. Filtering or weighting individual acquisitions prior to image analysis can overcome this problem.

## Abstract 2

Click here for author‐reader discussions

## 1 INTRODUCTION

Diffusion‐weighted imaging (DWI) is an essential component of multiparametric MRI (mpMRI)^1^, ^2^ and plays a critical role as the dominant sequence for identifying Prostate cancer,^3^, ^4^ which is the most common noncutaneous cancer in men in the United States.^5^ The “Prostate Imaging Reporting and Data System”^6^ is used by radiologists to analyze multiparametric MRI data and to assign cancer probability scores to regions of interest after the evaluation of DWI, T2‐weighted sequences, and dynamic contrast‐enhanced‐MRI.^2^

Apparent diffusion coefficients (ADCs), measured from DWI, are sensitive to cancers because the dense cellularity of cancers restricts water diffusion and thus increases the intensity of diffusion‐weighted signals.^7^ Chenevert et al. showed that ADC measurements are highly reproducible with variability less than 5% via standard phantom experiments,^8^, ^9^ demonstrating that MRI scanners, especially diffusion‐sensitizing gradients, provide excellent stability for very sensitive measurements. However, ADC measurements in patients are much more variable.^10^ A large component of this variability comes from motion artifacts.^11^, ^12^ Very small amounts of motions during diffusion‐encoding gradients can result in a significant loss of signal.^13^ Fluctuations in local magnetic fields also decrease the signal. This makes it more difficult to detect cancers. This signal loss is especially problematic for high *b*‐value DWI scans. Specifically, *b* values $\geq 900\;{s/\mathrm{mm}}^{2}$ produce significant phase dispersion. Therefore, DWI at high *b* values have low signal‐to‐noise ratio (SNR), and require multiple acquisitions. Clinical DWI scans are typically acquired with diffusion‐sensitizing gradients along three directions, with multiple acquisitions for each gradient direction.^14^ These acquisitions are reconstructed and then averaged together to increase signal‐to‐noise ratio and produce a composite image for diagnosis.^15^ This means that some signals that are corrupted by motion are combined with signals that accurately report the presence of cancer. This dilutes the signals that make cancers conspicuous. Gross motion between separate acquisitions can also cause errors, particularly in areas where tissue is heterogeneous. However, effects of this type of motion are expected to be much smaller than effects of microscopic motions, during diffusion‐sensitizing gradients.

Although it is widely accepted that DWI is subject to motion and other artifacts,^16^ there has been very little quantitative analysis of the directional and inter‐acquisition variability in prostate DWI. The study of Sadinski et al.^17^ shows that the standard deviation (SD) of repeated ADC measurements produced from *averaged* DWI scans is at least 10%. However, this underestimates the true variability because these estimates are produced from averages over multiple acquisitions and diffusion‐sensitizing gradient directions. This averaging masks the underlying variability.

We hypothesize that the distribution of per‐voxel signal intensity is skewed, and therefore conventional averaging is not optimal since it combines high signals on diffusion‐weighted images due to restricted diffusion with low signals that are due to motion (or fluctuations in local magnetic field). The method we propose to address this problem is based on the assumption that artifacts are more likely to *decrease* signal than to increase signal in DWI, especially at high *b* values. For example, with $b=900\;{s/\mathrm{mm}}^{2}$ or higher—highly localized motion of 25–50 microns due to the arterial pulse or peristalsis as well as gross motion (possibly due to breathing) can reduce or completely suppress signal. Some artifacts can increase signal, for example, gross motions that change the location of tissue between acquisitions, or changes in local susceptibility gradients between acquisitions that result in “signal pile‐up.”^18^ However, these effects tend to be less frequent and have less impact compared to local motion during diffusion‐encoding gradient pulses.^13^ The signal‐reducing effects of motion on DWI have been extensively studied in the MR literature for several other organs. Liau et al.^19^ investigated motion‐induced signal loss on liver DWI at $b=1000\;{s/\mathrm{mm}}^{2}$ and designed a method to favor high signals during averaging. Kwee et al.^20^ analyzed which direction of motion sensitizing gradients was most affected by cardiac motion on liver DWI. Another study suggested that “pseudo‐anisotropy” is an artifact that reduces the DWI signal and originates from anisotropic respiratory movement.^21^ In a more recent work, Hernando et al.^22^ showed that parts of organs closer to body motion are not evaluable on ADC maps due to severe signal loss in the DWI image which results in extreme overestimation in the ADC map. Aliotta et al.^23^ investigated the bulk‐motion signal losses occurring over the cardiac cycle on brain, liver, and heart DWI and showed how mean ADC values were significantly corrupted ($>3\times 10^{-3}{\text{mm}}^{2}/s$). Luna et al.^24^ studied the effects of motion on chest DWI and showed that motion artifacts related to breathing and vascular pulsation created low signal in all pulse sequences. Takahara et al.^25^ showed that signal intensity of small bowel contents can be suppressed due to normal peristalsis resulting in intraluminal turbulent flow. Metens et al.^26^ proposed optimized cardiac triggering for each acquisition during DWI of liver to reduce cardiac motion‐induced signal loss. Gurney et al.^27^ used a method very similar to the method presented here, based on the hypothesis that anomalously low signals reflect motion‐induced signal loss. They proposed an algorithm to reject these anomalously low signals in pancreas and liver DWI. This previous work is consistent with our assumption that high signals at high *b* values are more likely to correctly represent restricted diffusion, while low signals are more likely to result from artifacts in prostate DWI.

This study extends this previous work by introducing a statistical approach to quantitatively describe the level of variability in DWI over various protocols, patients and tissue types. In addition, we propose a novel editing method, “Editing for Restricted Diffusion (ERD)” to reduce the errors caused by acquisitions that are likely to be corrupted by signal loss due to local microscopic motions and/or fluctuations in local magnetic field. ERD is based on the principles introduced by Gurney et al. but extends it by incorporating the weighted sum of acquisitions, instead of sole filtering, which provides an enhanced contrast between healthy and cancer tissue.

## 2 METHODS

This study is a retrospective analysis of data from 10 patients who participated in two prospective studies that were compliant with Health Insurance Portability and Accountability Act of 1996 (HIPAA) and approved by the Institutional Review Board (IRB). Half of the patients in this study were imaged with a nonendorectal coil (NERC) protocol and the other half of the patients were imaged with an endorectal coil (ERC) protocol. More information on MR imaging and histologic analysis is provided in Supporting Information Appendix S1.

### 2.1 Analysis of directional and inter‐acquisition variability in DWI

#### 2.1.1 Signal deviation across acquisitions

First, we calculated the SD map of the signal intensities at the highest *b* value on a per‐voxel basis, across the multiple acquisitions. This part of the study was conducted to observe the effect of variation induced by the physiological motion of the prostate and to compare it with the theoretical Rician behavior. For this, we compared the variation at rectum, a region with no signal and little probability of motion‐induced signal loss—hence expected to follow a Rayleigh distribution (which is a special case of Rician distribution at signal‐to‐noise ratio = 0) versus the prostate tissue, which would follow a generalized Rician distribution, if the physiological signal loss is negligible.

We also compared the variability of the ADC values across acquisitions in cancer and healthy regions of interest (ROI) for ERC and NERC measurements. For cancer ROIs, we took the 3×3 voxel areas centered at the biopsy‐verified cancer location for each patient. For healthy ROIs, we used 3×3 voxel areas centered at a tissue that is verified by biopsy to be normal. We analyzed the inter‐acquisition variation via the distributions of ADC values, estimated over the acquisitions of the cancer and the healthy ROIs. We produced the ADC maps for each acquisition with multiple *b* value reconstructions using mono‐exponential least squares fit (1):

(1) $$D[i,j]=-\frac{\left|B|\underset{\forall b\in B}{\sum }b\ln(S_{b}[i,j])-\underset{\forall b\in B}{\sum }b\underset{\forall b\in B}{\sum }\ln(S_{b}[i,j]\right)}{|B|\underset{\forall b\in B}{\sum }b^{2}-{\left(\underset{\forall b\in B}{\sum }b\right)}^{2}},$$

where B is the set of *b* values used and |B| is the size of B, that is, number of different *b* values. $S_{b}[i,j]$ and D[i,j] are the diffusion signal intensity and ADC values of the voxel located at (i,j), respectively. Equation (1) yields the best fit for the mono‐exponential decay model $S_{b}[i,j]=S_{0}[i,j]\exp(-bD[i,j])$.

In addition, we calculated a distribution of the normalized ADC measurements, by scaling the ADC values within each voxel to vary in the range 0−1:

(2) $${\hat{D}}_{k}[i,j]=\frac{D_{k}[i,j]-D_{\min}[i,j]}{D_{\max}[i,j]-D_{\min}[i,j]},$$

where $D_{k}[i,j]$ is the ADC calculated from $S_{b}^{k}[i,j]$ (k th acquisition of diffusion signal) and $D_{\min}[i,j]$ and $D_{\max}[i,j]$ are the minimum and maximum ADC values for the voxel located at (i,j). Then, we measured the distributions of these normalized measurements within each 10‐percentile of the range of measurements. Such histograms are valuable to demonstrate the clusters of acquisitions within each voxel and observe the effects of inter‐acquisition variability, independent of the underlying ADC values within each voxel. The histograms per ROI are then calculated by summing the counts of normalized ADC values that fall within the same percentile bin. This histogram can be formulated as:

(3) $$\begin{array}{cc} & p_{r}=\underset{i,j\in \text{ROI}}{\sum }\overset{K}{\underset{k=1}{\sum }}1(0.01(r-1)<{\hat{D}}_{k}[i,j]\leq 0.01r),\\ & \;r=10,20,\cdots 100 \end{array}$$

where 1(A) is the indicator function that takes value 1 is A is true, and 0 otherwise.

#### 2.1.2 Inter‐direction variation analysis

We analyzed the variation within each diffusion‐encoding gradient direction and compared the inter‐direction and intra‐direction variation of the three directions. We performed this analysis to determine whether there is any pattern in the inter‐direction variation, perhaps due to anisotropic motion. Furthermore, we measured the variance of $S_{b}$ intensities of cancer voxels—calculated across the acquisitions per diffusion‐sensitizing gradient direction‐ and compared them with the Rician noise variance, to determine if the inter‐acquisition signal variation in a voxel could be explained by random electronic noise. The variations were displayed in radar plots where the edges of the radar plots show the variance of signal along each direction. To calculate the Rician noise variance, we first calculated sample variance over a 5×5 ROI on rectum ($\sigma_{R}^{2}$), a region with no signal (where the intensity variation is primarily due to random thermal/electronic noise generated by the lossy coupling between the detectors and the patient's body). The noise level for the Rician distributed high‐signal regions were then calculated as $N=\sigma_{R}^{2}(2-\pi /2)$ as shown by Gudbjartsson and Patz.^28^ We are aware that this may result in some errors; the noise level in the rectum is likely to be somewhat different from the noise in the prostate due to the distribution of the detectors and the effect of the SENSE reconstruction algorithm. Nevertheless, this protocol provides a rough estimate of the noise. This analysis of variation was done across several ROIs, including ROIs at the center and edge of the cancer as well as tissue surrounding the cancer.

Additionally, we conducted an analysis on the prostate region to quantify how much of the inter‐acquisition variation is caused by the inherent anisotropy of the water diffusion and how much of it is caused by the nonphysiological noise and motion‐induced signal loss. For this, we compared the inter‐directional variation, which gives a measure of anisotropy (measured as the range of variation between the means of signal along each direction) against the average intra‐direction variation (measured by the mean of the ranges of signal intensities of all acquisitions along each direction). The ratio of inter‐direction variability and the intra‐direction variability would give us a measure of how much of the inter‐acquisition variability can be explained by the intrinsic anisotropy of water diffusion. We note that the measurements used here to evaluate anisotropy do not provide an accurate calculation of anisotropy. This requires diffusion tensor calculations based on a larger number of gradient directions.^29^ Nevertheless, comparison of signals from multiple voxels acquired with different diffusion directions are sensitive to diffusion anisotropy.^30^

### 2.2 Editing for restricted diffusion

#### 2.2.1 ERD Filtering

We aim to suppress corrupted signals from individual acquisitions in an automated way, on a voxel‐by‐voxel basis. One straightforward way to do this is to apply a cutoff threshold as in Reference 27. We chose an ADC cutoff from the prostate MR literature, taking into consideration the scanner brand and scanning parameters. Chatterjee et al.^31^ observed the average ADC value on cancer ROIs to be $0.86\pm 0.18\times 10^{-3}{\text{mm}}^{2}/s$, measured over a cohort of 22 prostate cancer patients. Therefore, we picked the acquisition rejection cutoff at the upper limit of this range, that is, $1.04\times 10^{-3}{\text{mm}}^{2}/s$. The ERD filtering procedure acts on each voxel, compares the signal from each individual acquisition to the threshold and counts the number of acquisitions that fall below it, instead of averaging them. This provides a measure of consistency between the acquisitions and reduces the effects of artifacts. The ERD filtering is useful for (1) highlighting the regions with the most restricted diffusion for cancer detection, (2) identifying the amount of motion corruption by monitoring the number of individual signals that are filtered and similarly identifying voxels that have reliable signal because of few suppressed acquisitions, and (3) identifying the signals that can be used in an enhanced ADC calculation, as we present in the next section (ERD Weighting method) in a more generalized way.

#### 2.2.2 ERD weighting

In addition to the ERD filtering described above, which highlights the regions with the most restricted diffusion, agnostic of the occasional motion‐corrupted signals, we also implemented a weighted average of acquisitions instead of the conventional averaging. This yields an ADC map with enhanced contrast. Instead of hard rejection, this method follows the assumption that artifacts are more likely to decrease signal than to increase signal in high *b* DWI^19^, ^20^, ^23^, ^27^ and attaches weights to the signals from multiple acquisitions prior to combination of individual signals, suppressing the effect of low signals. These sets of weights are calculated for each voxel using the soft‐max formula in (4):

(4) $$w^{k}[i,j]=\frac{\exp(\frac{S_{b}^{k}[i,j]}{\tau })}{\overset{K}{\underset{j=1}{\sum }}\exp(\frac{S_{b}^{j}[i,j]}{\tau })},$$

where $S_{b}^{k}[i,j]$ is the k th acquisition of the high *b* signal intensity at voxel located at (i,j) and τ is the “temperature” parameter. The set of weights $w^{k}$ for each voxel sums to one, making it operate like a probability mass function. The enhanced DWI is then calculated as the weighted sum of the acquisitions as shown in (5).

(5) $${Ŝ}_{b}[i,j]=\sum_{k=1}^{K}w^{k}[i,j]S_{b}^{k}[i,j].$$

The soft‐max function gives more weight on the acquisitions with high signals. If some acquisitions are significantly lower than the acquisition with the highest signal, their weights are suppressed. The functionality of the “temperature” parameter τ is two folds: first, it controls how steep this “high‐signal favoring” is. As τ goes to infinity, the set of weights would approach the uniform distribution, which would be equivalent to the conventional signal averaging in DWI. Lower τ values would suppress the low signal more drastically, favoring the acquisition with the maximum signal value as it approaches to zero, hence the name *soft‐max*. One other role of the τ parameter is that it inherently enhances the contrast between the cancer and healthy tissue: Since the signal on a voxel with more restricted diffusion, that is, cancer, is higher; the relative intensity of τ becomes lower, hence $w^{k}$ favors the higher signals more. On the other hand, the same τ will appear relatively larger for voxels with low signals and the weighted sum of the acquisitions will approach the conventional signal averaging on such voxels. We also employed the ERD‐weighting method on multiple acquisitions of a water phantom scanned at 18°C to evaluate if the method incorrectly reduces the ADC values of known subjects.

## 3 RESULTS

### 3.1 Inter‐acquisition variability in DWI

**FIGURE 1:**
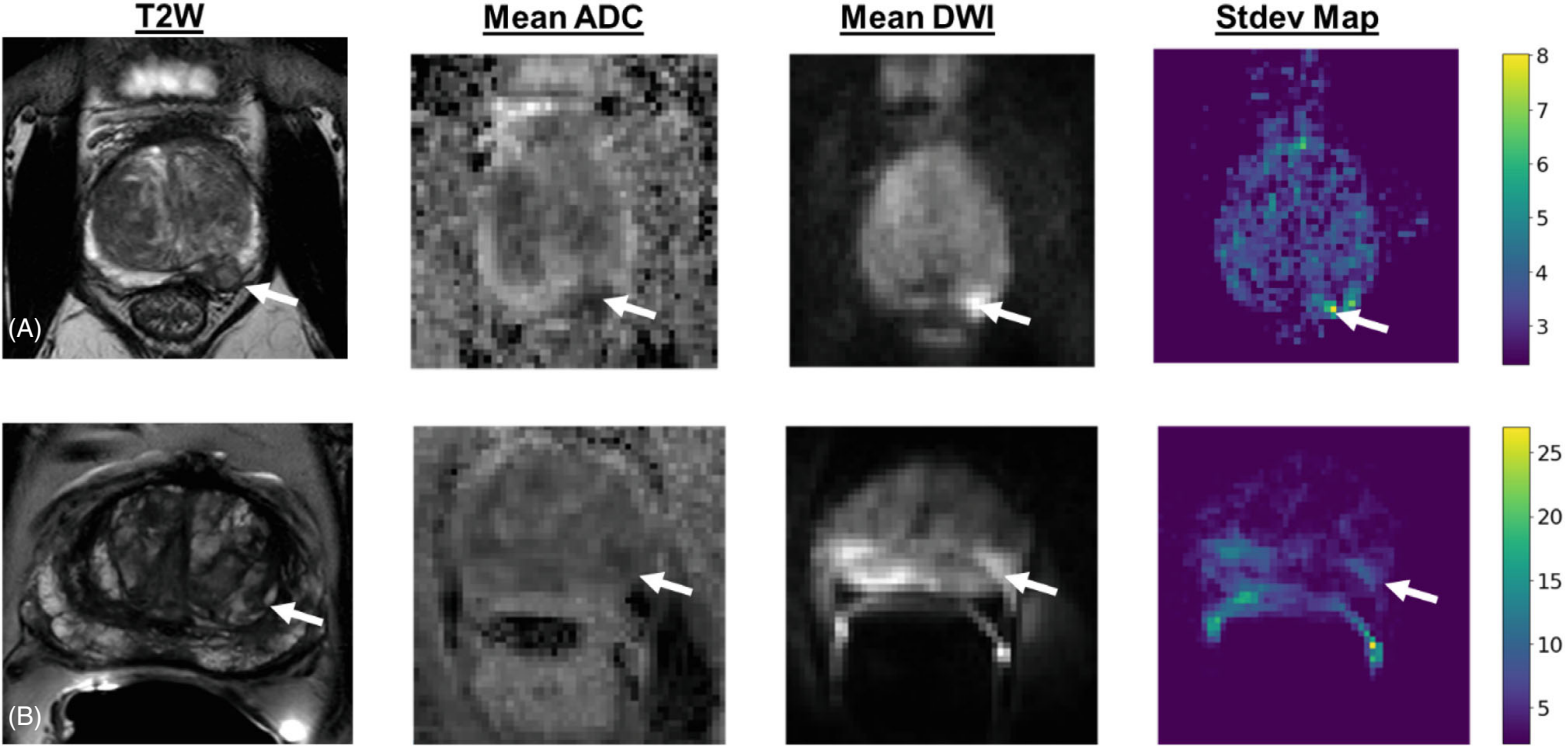
T2W, mean apparent diffusion coefficient map, mean diffusion‐weighted image (DWI) and corresponding DWI standard deviation for (A) a nonendorectal coil and (B) an endorectal coil patient

Figure 1 shows T2, ADC, and diffusion‐weighted images at highest *b* value, and the SD maps for a NERC and an ERC scan. The NERC patient has GS3+4 and the ERC patient has GS4+3 cancers. The SD map was windowed with the minimum value as the Rician SD value to demonstrate the variation introduced by the physiological noise. The nonphysiological component of the noise was calculated from the ROI at the rectum. Since the SD of the high signal regions should be upper bounded by the ${\left(\sqrt{2-\pi /2}\right)}^{-1}$ times the SD of the rectum measurements,^28^ the variation that is visible on the SD map reflects the increase that is mostly induced by local motion or magnetic field variations. Moreover, the SDs in regions with more restricted diffusion are higher than the SDs in most of the rest of the prostate, while the SD of nonphysiologic (thermal/electronic) noise is independent of signal amplitude. More analysis of the effect of motion‐induced signal loss on the signal distribution can be found in Supporting Information Appendix S1 and Figure S1.

**FIGURE 2:**
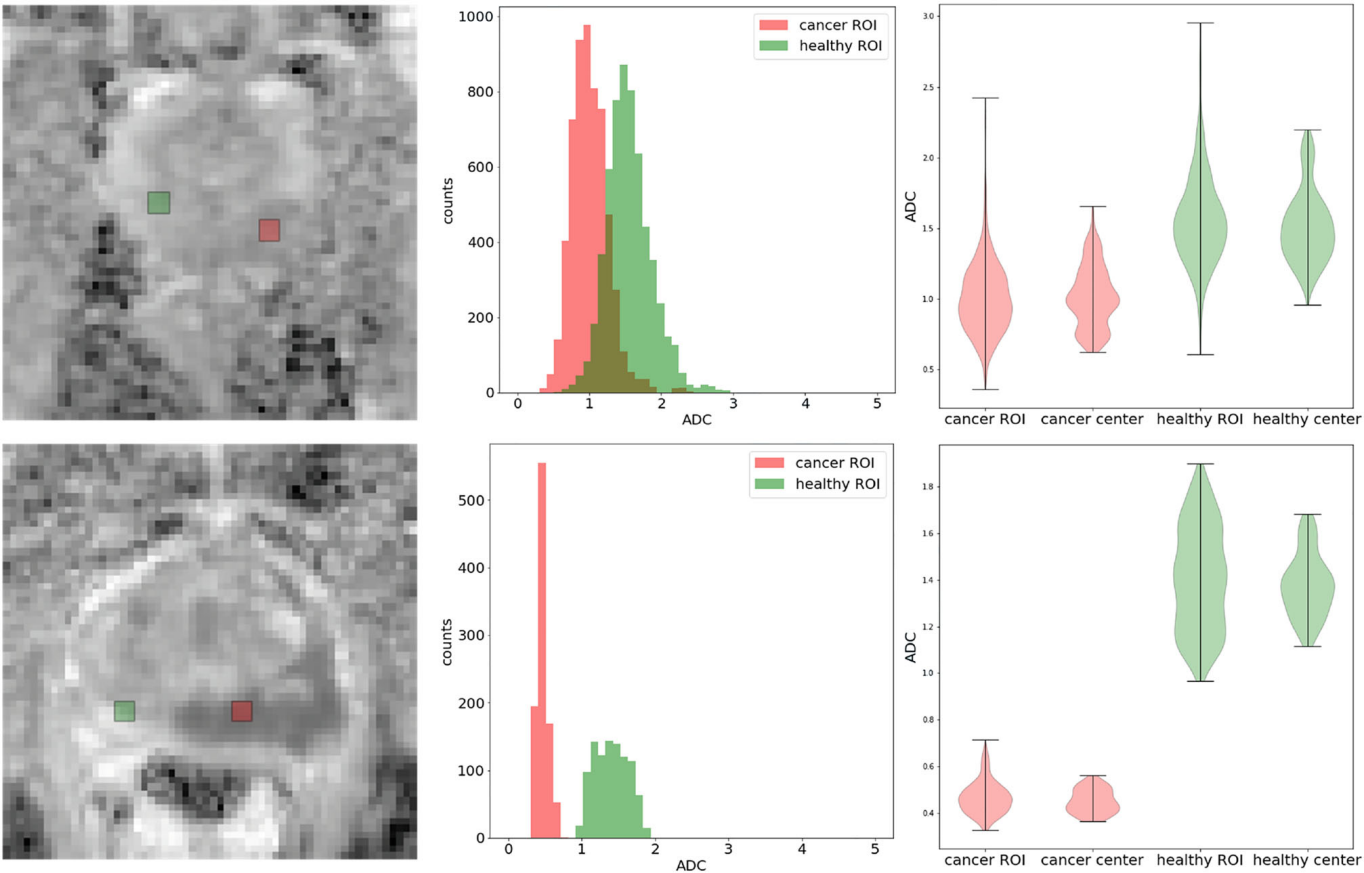
Histograms of apparent diffusion coefficient (ADC) values calculated on the cancer (red) and healthy (green) ROIs for a nonendorectal coil (NERC) (top) and a endorectal coil (bottom) patient. The corresponding ADC maps and the cancer/healthy ROIs are also provided. Violin plots on the right compare inter‐acquisition variation against inter‐voxel variation caused by spatial heterogeneity.

Figure 2 shows histograms of ADC values for two patients. The histograms on the middle figure are calculated using all least squares fits for all combinations of acquisitions and all *b* values. NERC scans are affected by the inter‐acquisition variation more than ERC, which is consistent with the results discussed above. The histograms for cancer ROIs (red color) and healthy ROIs (green color) overlap considerably for NERC cases. There is much less overlap for ERC cases. The NERC histograms are unimodal but significantly skewed towards high ADCs. The violin plots on the right show the variation of ADC values calculated using the acquisitions on the center voxel of the cancer/healthy ROI. This variation is compared to the variation over the whole ROI, which was calculated using all acquisitions for all voxels within the ROI. This demonstrates that most of the variation comes from the inter‐acquisition variability, rather than the variation across the voxels (spatial variation).

**FIGURE 3:**
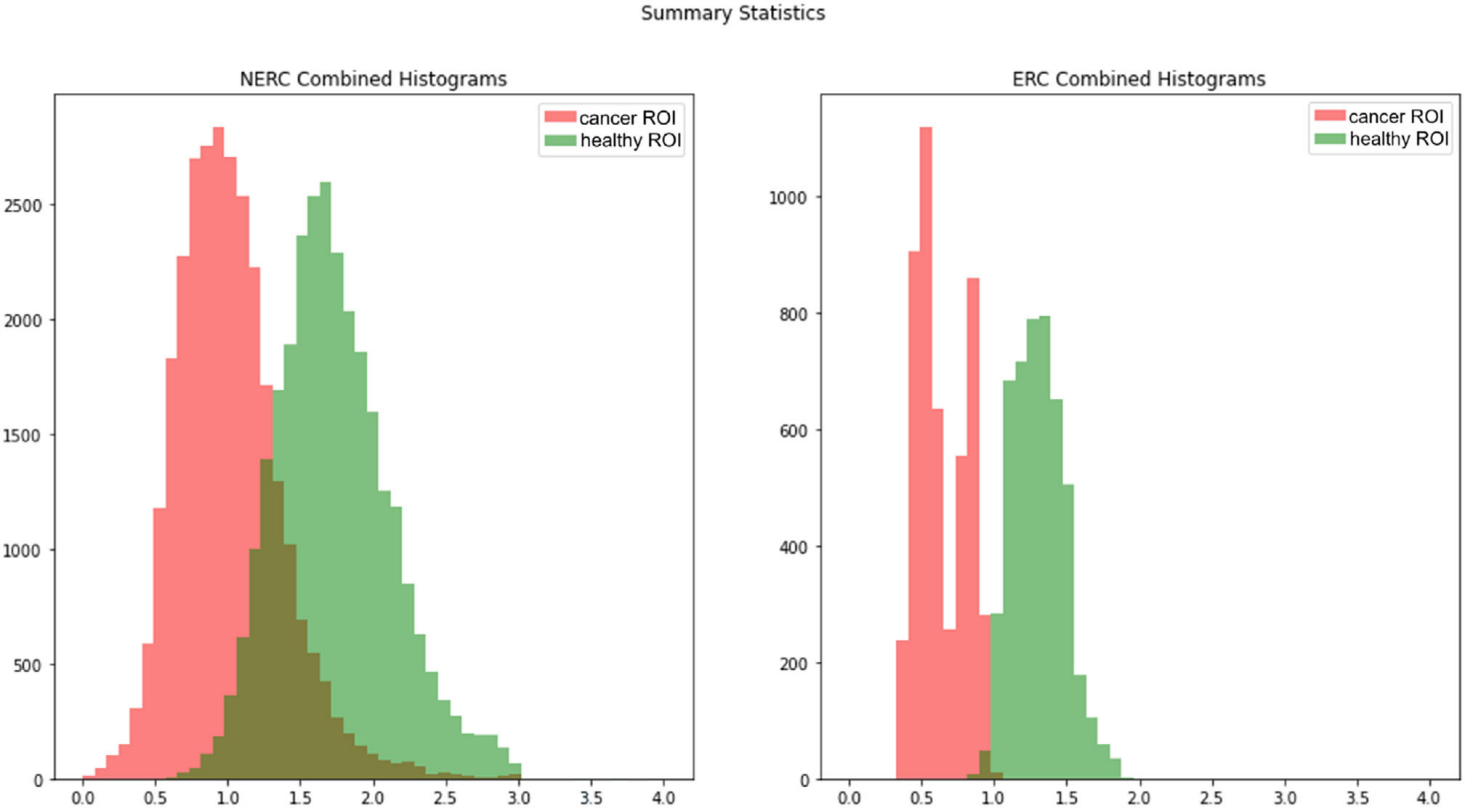
Histograms of cancer (red) and healthy (green) apparent diffusion coefficient values for nonendorectal coil (NERC) (left) and endorectal coil (right) patients. The “olive green” color indicates the region where the tail of the cancer distribution overlaps with the healthy distribution.

These features are also apparent in the histograms showing combined data from all individual acquisitions (Figure 3). The adverse effects of inter‐acquisition variation are much stronger for NERC versus ERC. Both protocols exhibit positive skewness, as would be expected for motion artifacts. Skewness was calculated to test whether the bulk motion significantly decreases DWI signal intensity (increases ADC). This indicates that the population mean of all acquisitions for each b‐value may not be a good estimator of the correct value. When signals from all acquisitions are combined to produce the final signal intensity in each voxel, this “skewness” decreases the contrast for cancer detection. Moreover, both the healthy and cancer ROIs from NERC scans have large kurtosis values, indicating a more peaked and wider‐tailed distribution than a Gaussian distribution. For ERC scans, the kurtosis values were very close to Gaussian's kurtosis value of 3.0. Moreover, a large and positive kurtosis value indicates that the tails of the distribution of variation exceed the tail of a Gaussian with the same mean and SD. This emphasizes the need for ERD, that is, eliminating acquisitions with high probability of motion‐induced signal loss, prior to mean signal estimation. The statistics are given in Table 1.

**TABLE 1:** Summary statistics of apparent diffusion coefficient (ADC) values for cancer and healthy 3×3 ROIs

		NERC Cohort		ERC Cohort		Phantom	
		DWI	ERD	DWI	ERD	DWI	ERD
ADC ($\times 10^{-3}{\text{mm}}^{2}/s$)	Cancer	0.98 ± 0.21	0.77 ± 0.22	0.63 ± 0.18	0.58 ± 0.20	—	—
	Healthy	1.76 ± 0.21	1.65 ± 0.23	1.39 ± 0.13	1.29 ± 0.13	1.94 ± 0.00	1.94 ± 0.00
Skewedness	Cancer	1.02 ± 0.2	—	0.03 ± 0.5	—	—	—
	Healthy	1.00 ± 0.3	—	0.17 ± 0.2	—	0.28 ± 0.00	—
Kurtosis	Cancer	5.94 ± 2.9	—	2.92 ± 0.3	—	—	—
	Healthy	5.05 ± 1.4	—	2.67 ± 0.4	—	2.54 ± 0.00	—

Abbreviations: DWI, diffusion‐weighted image; ERC, endorectal coil; ERD, editing for restricted diffusion; NERC, nonendorectal coil.

**FIGURE 4:**
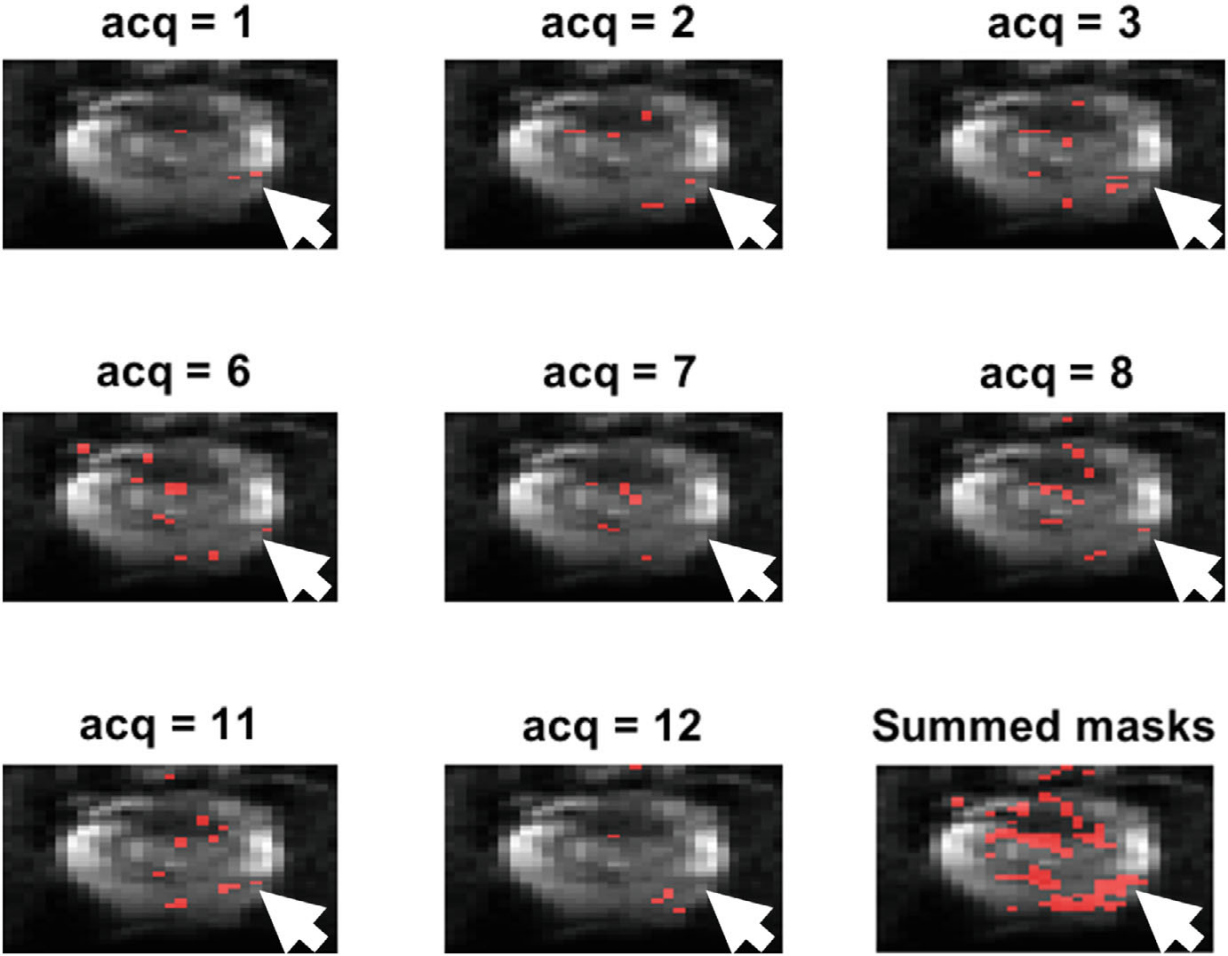
Visualization of inter‐acquisition variability over 8 of the 24 independent acquisitions of a nonendorectal coil patient, along with the map of the union of detections from all acquisitions. Red voxels show areas where the apparent diffusion coefficient is below the diagnostic threshold of $1.04\times 10^{-3}\;{\text{mm}}^{2}/s$ and the arrow shows the location of the biopsy‐verified cancer.

Figure 4 demonstrates the inter‐acquisition variability over the independent acquisitions of a NERC scan for $b=900\;{s/\mathrm{mm}}^{2}$. The figure shows the voxels (red) that have ADC values below $1.04\times 10^{-3}\;{\text{mm}}^{2}/s$ for 8 of the 24 $S_{b}$ acquisitions, and additionally the union of all such detected voxels over all acquisitions. The figure with all of the 24 acquisitions is provided in Supporting Information Figure S22. Voxels within the cancer ROI are sometimes above and sometimes below the cutoff. There are also some false positive areas where signal is above the cutoff. Some individual acquisitions show more restricted diffusion in some of the cancer voxels and other acquisitions show more restricted diffusion in some other cancer voxels. Furthermore, for some acquisitions the signal is completely lost, and these acquisitions show almost no restricted diffusion within the cancer.

**FIGURE 5:**
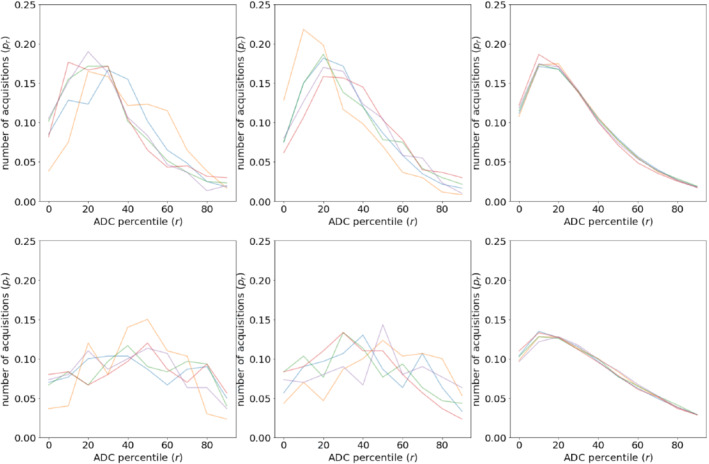
Distributions of the acquisitions on min‐max normalized apparent diffusion coefficient ranges calculated on cancer (left), healthy (middle) and all prostate (right) voxels. Each patient is shown with a different color. Nonendorectal coil (NERC) is on the top and endorectal coil (ERC) is on the bottom. Note that the normalized distribution appears flatter for ERC, because the SD and the range in $D_{\max}-D_{\min}$ are lower for ERC, but this range is being stretched to the same scale as NERC, that is, 0–1.

Figure 5 shows the min‐max normalized distributions of ADC values calculated along the multiple acquisitions, as shown in Equation (3). The histograms are demonstrated as line graphs so that the differences of histograms of different patients can be observed. Top row shows the five NERC patients with different colors, and the bottom row shows the ERC patients. The histograms on the left and middle were calculated on the cancer and healthy ROIs, respectively. The histograms on the right show the averages of histograms of all prostate voxels. The effects of inter‐acquisition variation exhibit a similar pattern for all patients; the majority of the values for independent acquisitions cluster at lower values but also extend to the higher values due to motion‐induced signal loss. For NERC, where this effect is stronger than ERC, the majority the of the acquisitions (51%) are clustered on the first 1/3 of the range of the acquisitions with the peak at smaller values. However, a significant number of acquisitions yield ADCs at higher values. This tends to skew the calculated average ADC to higher values. For ERC, effect of inter‐acquisition variation is less dramatic, and the distribution is flatter. This is due to the fact that the unnormalized range of variation in ERC is smaller than NERC as seen on Figure 3. Still, many of the signals are in the first 30 th percentile.

### 3.2 Inter‐direction variability in DWI

**FIGURE 6:**
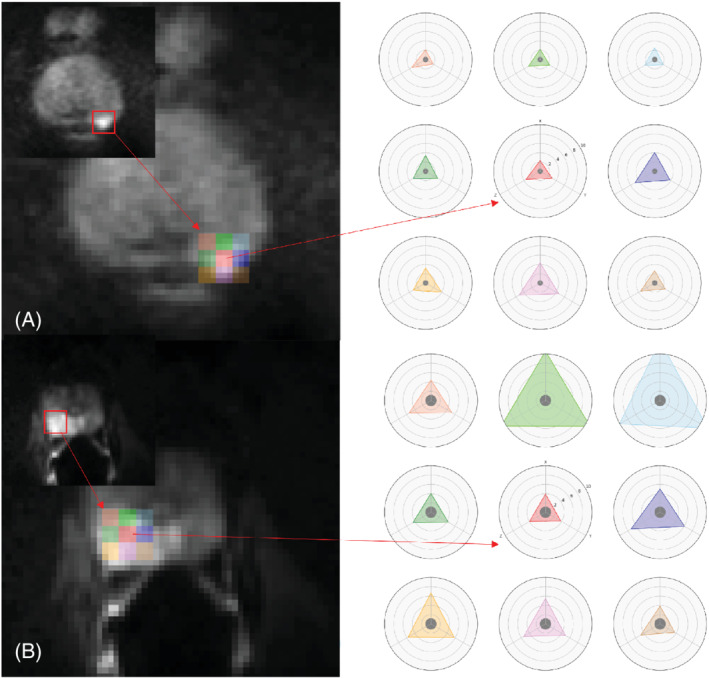
Radar plots demonstrating the inter‐direction and inter‐acquisition signal variation for (A) a nonendorectal coil and (B) an endorectal coil patient, calculated at the center and the edges of cancer region. The variances of signal intensity were calculated at the centers of the colored ROIs. The extent of the triangles' corners depicts the signal variation for each direction. The radius of the gray circle in the middle shows the Rician noise variation. Note that the fact that the triangles are larger than the noise shows that the inter‐acquisition variation in cancer is much larger than the noise, which is consistent with the SD map in Figure 1. Moreover, the fact that the triangles are far from being equilateral shows the inter‐direction variability.

The “Radar” plots in Figure 6 show the variance of signal values across acquisitions and diffusion‐encoding gradient directions for the voxels indicated in the image. There are nine radar plot/triangles with different colors and each triangle represents the inter‐direction variation of the voxel at the center of that ROI with the same color as the triangle. The small dark circle at the centers of the triangles shows the estimated variance of the Rician noise. The distance from each vertex to the center of the “noise circle” represents the variance of signals acquired for the voxel for each diffusion gradient direction (four different acquisitions for ERC images and eight different acquisitions for NERC images). Inter‐acquisition and inter‐direction variation are summarized by size and asymmetry of the triangles. The large area of the triangle relative to the area of the circle representing noise shows that signal variation is much too large to be explained by Rician noise. The asymmetry of the triangles demonstrates variability between diffusion‐sensitizing gradient directions associated with each image voxel. There was no consistent trend, that is, the diffusion‐sensitizing‐gradient direction with lowest variability was not consistent between voxels or patients.

The intra‐direction versus inter‐direction variability ratio, employed to observe how much of the inter‐acquisition variation can be explained by the anisotropic motion artifacts was less than 0.20 on average. This suggests that only a small portion of the inter‐acquisition variability can be explained by the inter‐direction variability.

### 3.3 Editing for restricted diffusion

**FIGURE 7:**
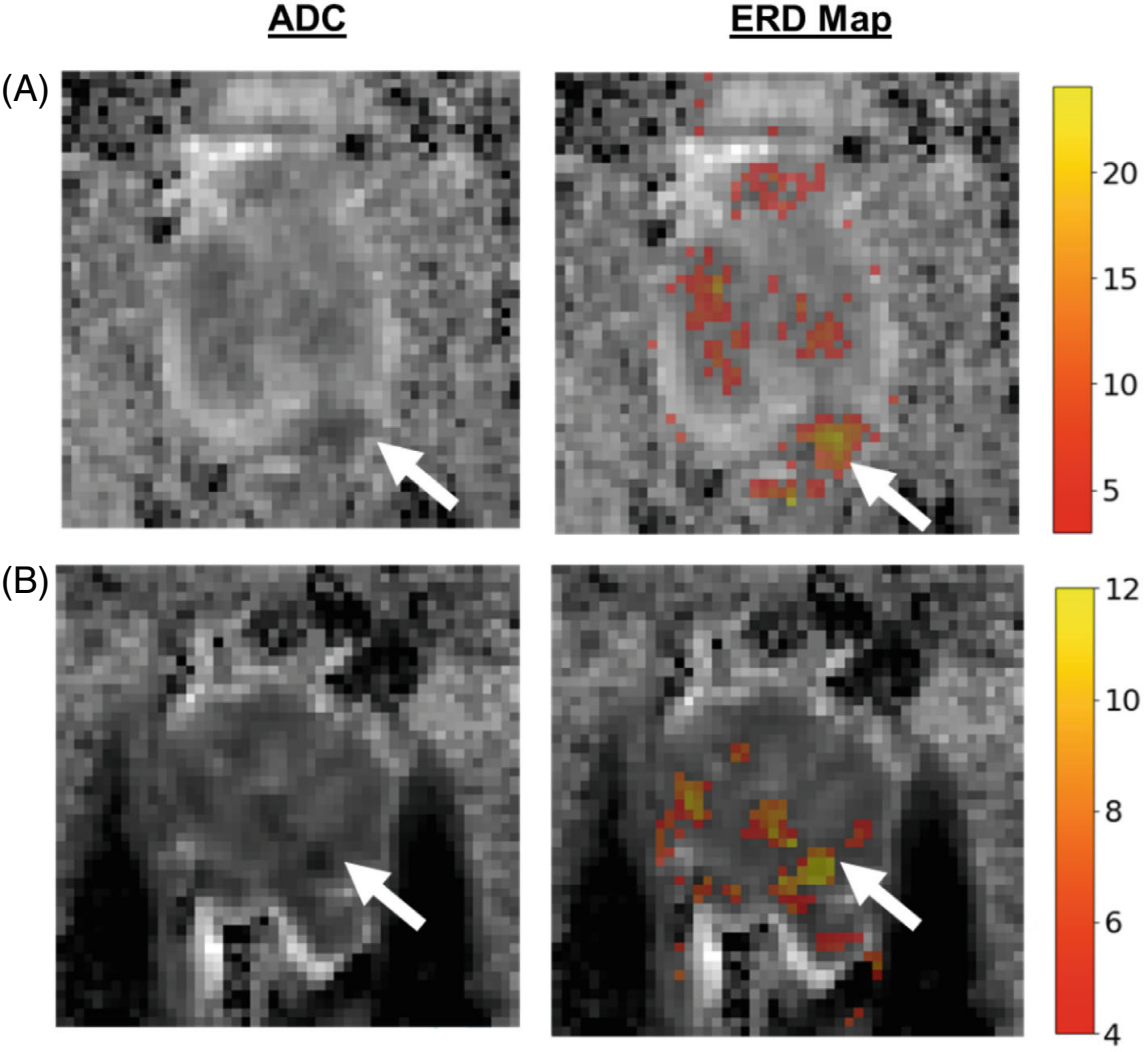
Examples of editing for restricted diffusion, for (A) a nonendorectal coil patient (GS4+3) and (B) an endorectal coil patient (GS3+4). The areas with restricted diffusion are depicted with the colored overlay. The colors of the heatmap indicate the number of acquisitions that exhibited more restricted diffusion. Arrows point to the biopsy‐verified cancers.

We applied ERD to 10 cancer targets. For filtering, we applied the ERD threshold $1.04\times 10^{-3}{\text{mm}}^{2}/s$, which was taken from Reference 31 to highlight the areas with the most restricted diffusion. Examples of ERD output are shown in Figure 7, for a NERC and an ERC patient both with Gleason 7 cancers. The ERD filter highlights (color overlay) the marked PZ cancers and also identifies benign TZ (possibly benign prostatic hyperplasia‐BPH) areas. The color of the marks shows the number of acquisitions for each voxel that exhibit more restricted diffusion as given in the heatmap. For example, the yellow color means that all of the acquisitions for the corresponding voxel indicate more restricted diffusion, whereas red indicates that some of the acquisitions do not have ADC values that are consistent with restricted diffusion. This shows that, in this example, more acquisitions for each BPH voxel are rejected by ERD compared to cancer voxels. ERD filtering results along with the biopsy‐verified cancer/healthy ROIs for all patients in the study are given in the Supporting Information Figures S2–S11.

The false positive rates of ERD were calculated with a two‐fold analysis—one based on the healthy ROI on the cancer slice, and another based on the whole prostate area on a slice that is 2 cm superior to the cancer slice. We applied the ERD cutoff threshold to the voxels in these two apparently healthy ROIs and measured the percentage of voxels for which more than 50% of the acquisitions exhibit more restricted diffusion (i.e., below the ERD threshold). In the healthy ROI, the false positive rate was zero and on average 2.3% and 5.9% of all acquisitions were below threshold, for NERC and ERC patients, respectively. In the other ROI, which involves a slice of the whole prostate with no verified cancer, the false positive rate was 3.2% and 4.7% for NERC and ERC, respectively.

**FIGURE 8:**
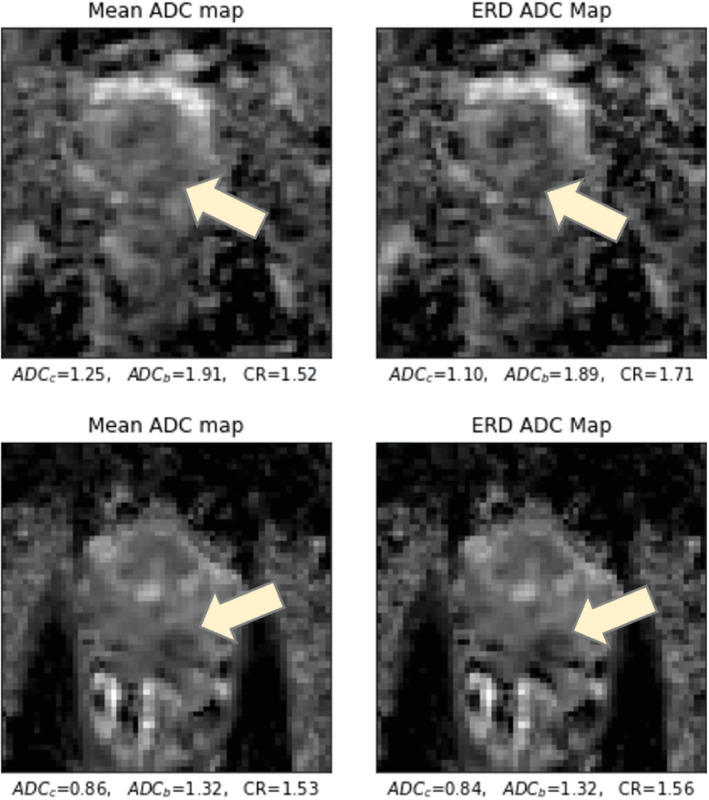
Examples of editing for restricted diffusion weighting for a nonendorectal coil patient (top) and an endorectal coil patient (bottom)

**FIGURE 9:**
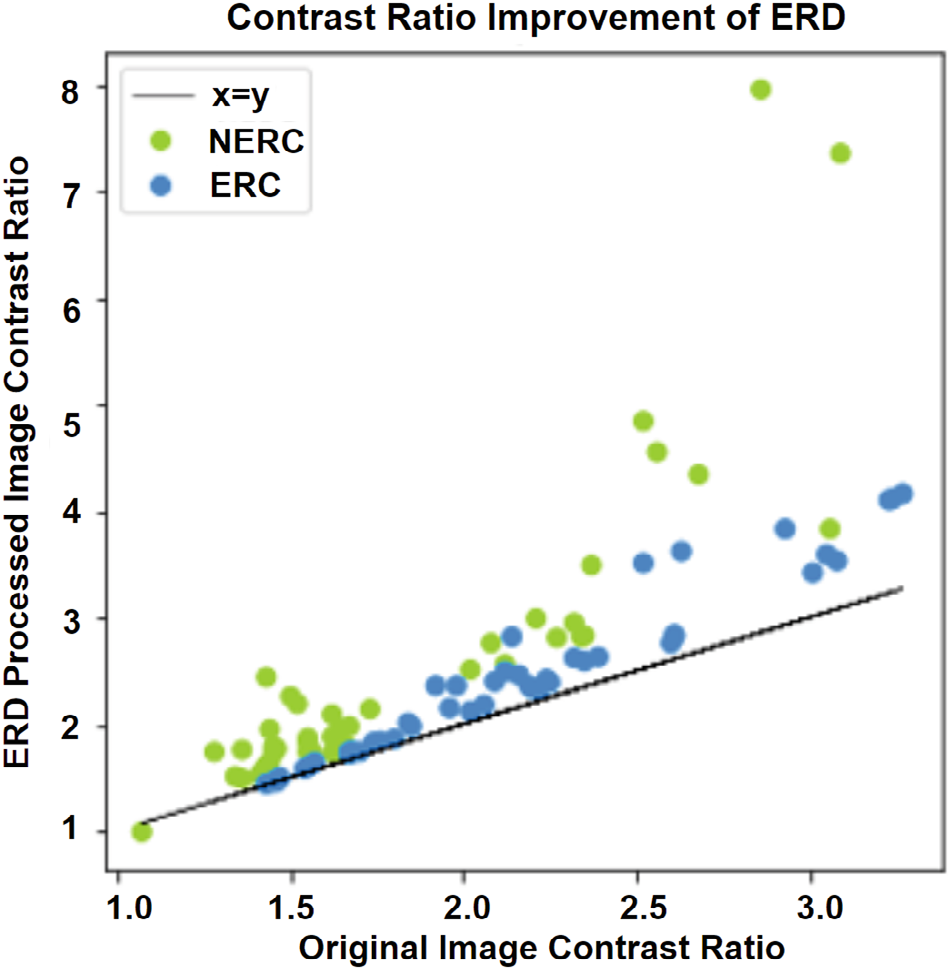
The scatter plot of the contrast ratio improvement over the studied cancer patients

When the soft‐max weighting given in Equations (4) and (5) was applied to the high *b* images, the contrast ratio between the cancer and healthy ROIs on ADC images improved 30.7% on average for NERC patients and 13.0% on average for the ERC patients. Examples of the ADC maps obtained with the conventional averaging of DWI acquisitions and the ADC map obtained by the ERD weighting are provided in Figure 8. ERD‐weighted ADC maps, along with the biopsy‐verified cancer and healthy ROIs for all other patients are provided in Supporting Information Figures S12–S21. The contrast between the cancer and healthy tissue improves for all of the patients after the ERD weighting. Table 1 also shows that the ERD weighting decreases the ADC values on cancer regions more than in regions of healthy tissue, which explains the improvement in the contrast. This suggests that, as expected, signals from water with more restricted diffusion are more sensitive to microscopic motions during diffusion‐sensitizing gradients than more freely moving water. Also, application of ERD on water phantom did not change the ADC value. The scatter plot demonstrating the improvement in the contrast ratio, calculated over unprocessed and processed images, is given in Figure 9. Figure 9 shows that while contrast is improved for almost all of the cancers, by far the greatest improvement is achieved for NERC images.

## 4 DISCUSSION AND CONCLUSIONS

We analyzed the individual acquisitions that are used to produce averaged DWI and ADC maps. We also measured the variability associated with each diffusion‐encoding gradient direction. The variability is much larger than the electronic noise level, and large compared to the expected difference between cancer and normal tissue, especially for NERC images. For NERC images, the distributions of ADC values for cancer and healthy tissue overlap significantly. On average, 43.4% of the ADC values for each voxel in cancer ROIs calculated from individual high *b* value acquisitions fall into a range that is much larger than the expected values for malignant tissue. When these signals are combined with other acquisitions that show more restricted diffusion in the same voxel, the cancer signal is obscured. The ADC histograms are skewed toward higher values, especially for NERC images. This is consistent with the hypothesis that many of the signals acquired for each voxel are corrupted by local motion. This variability and skewness of the distribution makes it difficult to distinguish between healthy and cancer tissue. The measured ADCs are likely much higher than the true ADCs.

These results are consistent with a priori expectations. Motion during diffusion‐encoding gradients that produce many cycles per millimeter in the phase of magnetization are expected to have a larger effect on signal intensity than motion between acquisitions. The primary source of variability measured in this study is very local—there is little correlation between changes in signal intensity in neighboring voxels. More work will be required to identify the sources of variability.

The data show that ERC images have far fewer motion artifacts than NERC images. Histograms of the signal for each voxel measured in separate acquisitions in cancer and healthy ROIs show better separation between cancer and healthy tissue for the ERC protocol. The results suggest that the ERC is stabilizing the rectal wall—and reducing the motions that are primarily responsible for the broader histograms in NERC cases. The higher “b” value for the ERC acquisitions, combined with higher signal‐to‐noise ratio also helps to distinguish between cancer and healthy tissue, but does not explain the narrower histograms for ERC. Since NERC imaging is likely to be the method‐of‐choice for mass screening for prostate cancer, it will be necessary to address the differences between ERC and NERC imaging. ERD may be an effective solution and one example of such a method is shown here.

The study described here is different from previous published measurements of variability of DWI in that we investigate the effects of inter‐acquisition variation and evaluate the efficiency of conventional averaging. The results support the hypothesis that with multiple acquisitions contributing signal to each voxel in cancer ROIs, cancers are clearly shown by some of the acquisitions, and not shown by others.

ERD effectively conserves signal from areas of the most restricted diffusion, for example, cancers. ERD weighting was applied by giving less weight to motion‐corrupted signals. This resulted in lower ADCs for any voxel with restricted diffusion, including cancers and BPH. The soft‐max weighting with the τ parameter gives more weight to acquisitions with high signals, even if many of the acquisitions are corrupted by motion. Furthermore, on the areas with less‐restricted diffusion, the function gives a more uniform set of weights across the acquisitions. The improvement in cancer to prostate contrast ratio demonstrates this functionality. Another observation is that the tissue with more restricted diffusion (e.g. in cancers) has more sensitivity of the diffusion‐weighted signal to microscopic motion than the tissue with less restricted diffusion (e.g., in normal prostate). This means that methods that minimize motion artifacts have a greater effect on signals from cancers versus signal from healthy tissue.

## 5 CONCLUSION

Evaluation of the multiple independent acquisitions that contribute to the signal in each voxel in the final diffusion‐weighted image allowed us to evaluate variability in high *b* value images. We showed that the distribution of signals from multiple acquisitions is skewed and not Gaussian. Therefore, combining all of these signals with conventional averaging obscures the cancer. The analysis demonstrated here can assess whether the signal from individual voxels is a marker for cancer. ERD can guide radiologists to place more or less emphasis on DWI data for cancer diagnosis, depending on the level of variability measured. Although this report focuses on conventional DWI of the prostate, the methods can be applied to MRI of other tissues and organs in the body where motion artifacts or fluctuating magnetic field gradients are a concern. Future versions of this method will use unsupervised machine learning techniques working on different acquisitions, and other information to reduce false positives while further increasing sensitivity to cancer.

## CONFLICT OF INTEREST

Ajit Devaraj is affiliated with Philips Research North America. Aritrick Chatterjee, Aytekin Oto and Gregory S. Karczmar report equity in QMIS LLC, outside the submitted work.

## Supporting information


**Appendix S1**. Supporting information
