# Supplementary material for: Directional and inter‐acquisition variability in diffusion‐weighted imaging and editing for restricted diffusion
Source: Magn Reson Med. 2022 Jul 21;88(5):2298–310. doi: 10.1002/mrm.29385 (PMC9545544; doi:10.1002/mrm.29385)
Supplement: Supplementary file 1 — Appendix S1. Supporting information [file MRM-88-2298-s001.docx]

**SUPPLEMENTARY MATERIALS**

1. **Computational Materials**

The analyses were done in Python version 3.8.8. For histogram calculations and visualizations the matplotlib (v3.3.4) and scikit-learn (v0.24.1) libraries were used.

1. **MR imaging and Histologic analysis**

Patient scan dates ranged from 07/2020-08/2021. Patients with elevated PSA with known or suspected prostate cancer were recruited and provided informed consent prior to imaging. The cancers analyzed in this study were confirmed by biopsy or prostatectomy. Patients were scanned on a 3T Philips Ingenia MRI scanner with an anterior 16 element phased detector array, and a 12-element posterior phased detector area. 5$patients were scanned with NERC protocol and 5 patients were scanned with ERC protocol in addition to the phase arrays mentioned above. DWIs were acquired at multiple b values with highest b-values of either b=900 s/mm^2^ (non-endorectal coil, 8 acquisitions per diffusion encoding gradient direction) or b=1500 s/mm^2^ (endorectal coil, 4 acquisitions per diffusion-encoding gradient direction). The details of the DWI sequences are given in the Supporting Information Table S1. Raw k-space data was exported from the scanner and reconstructed using Reconframe (Gyrotools, Zurich, Switzerland) into separate images for each acquisition.

For patients who received biopsies, 12 cores were biopsied using the standard random TRUS protocol, and in addition, some biopsy targets were identified by a radiologist based on PI-RADS 2.1. For patients who received prostatectomy, whole-mount tissue sections were prepared. Prostate tissue was then analyzed for prostatic adenocarcinoma and assigned a Gleason Score (GS) by an experienced pathologist.

**Table S1: Experimental setup in DWI reconstuction**

**
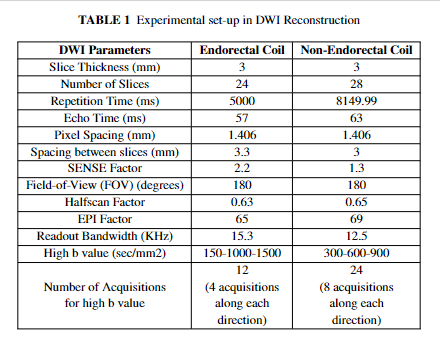
**

1. **Analysis of non-physiological noise:**

We analyzed the effect of motion-induced signal loss on the signal distribution. For this, we measured the distribution on three regions of interest: (1) rectum, which is Rayleigh distributed with positive skewness, (2) prostate area, which is Rician distributed and has a skewness very close to zero, and additionally the cancer region, which is consistent with the motion-induced signal loss added to the non-physiological noise resulting in a negatively skewed distribution. The samples of these distributions can be found in the Supporting Information Figure S1. The histogram of the area on the rectum (left) exhibits Rayleigh distribution with positive skewness. The distribution of the prostate area (middle), where there is signal is Rician distributed, has a skewness very close to zero. On the other hand, the distribution on cancer region (right) tends to reflect the effects of an additional signal loss, in addition to the non-physiological noise and makes the distribution negatively skewed.


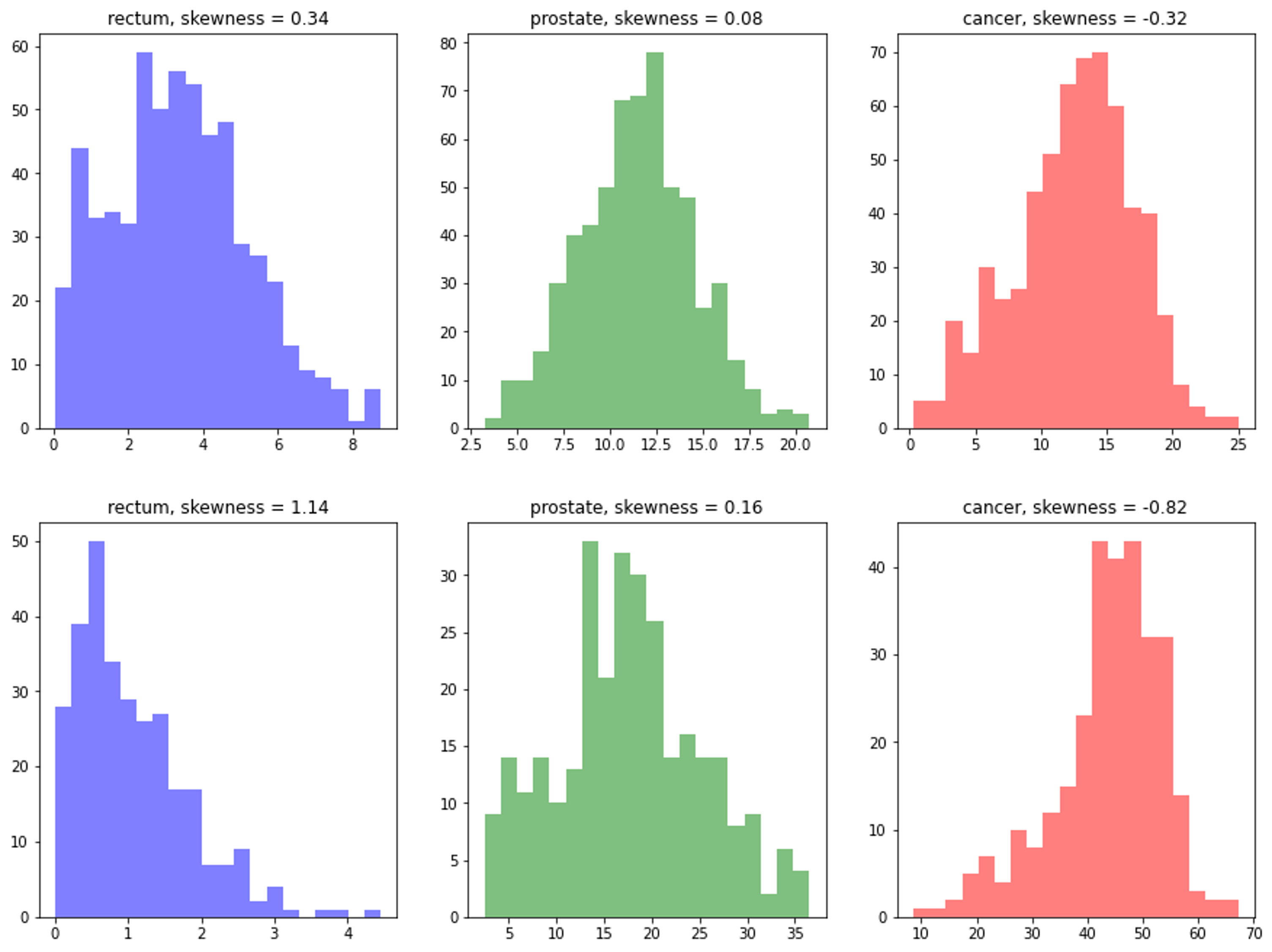


Figure S1: Signal distributions of different ROIs, rectum (left), healthy prostate (middle), cancer region (right), demonstrating the effect of motion-induced signal loss on.

1. **ERD FILTERING RESULTS FOR ALL PATIENTS:**

Color map of the number of surviving acquisitions are arranged as thus:

1. If more than half of the acquisitions for each voxel is above the ERD threshold, no marker is shown.
2. Red marker is shown for voxels with more than half and less than 80% of the acquisitions exhibit restircted diffusion.
3. Yellow marker is shown for voxels with more than 80% of its acquisitions exhibit restricted diffusion.


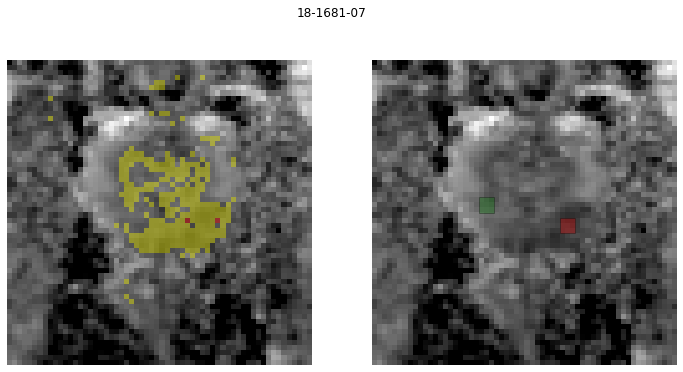


Figure S2: ERD Filtering for the NERC Patient 07.


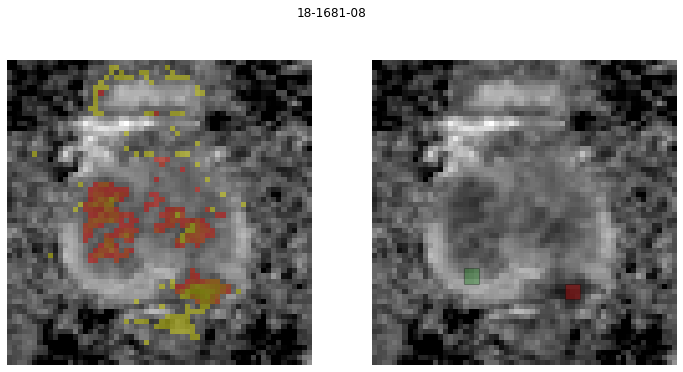


Figure S3: ERD Filtering for the NERC Patient 08.


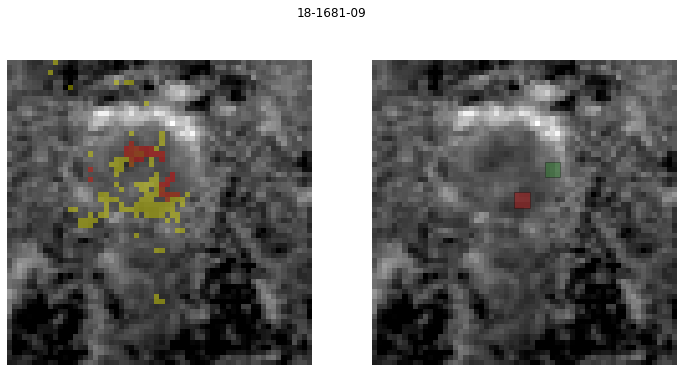


Figure S4: ERD Filtering for the NERC Patient 09.


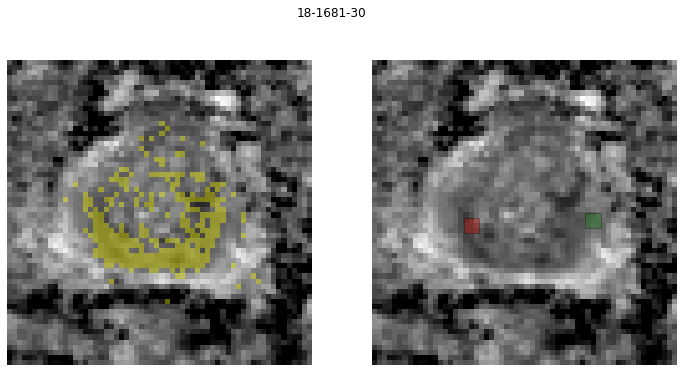


Figure S5: ERD Filtering for the NERC Patient 30.


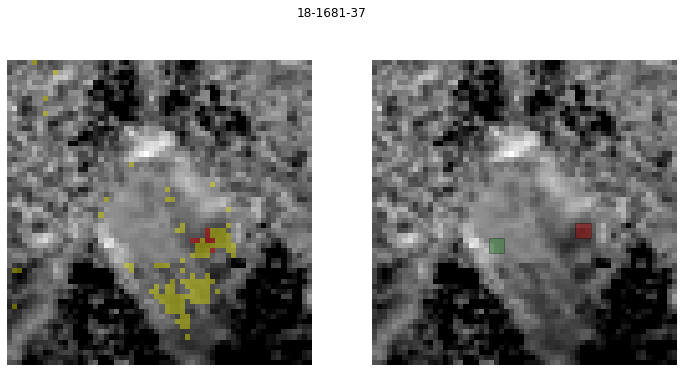


Figure S6: ERD Filtering for the NERC Patient 37.


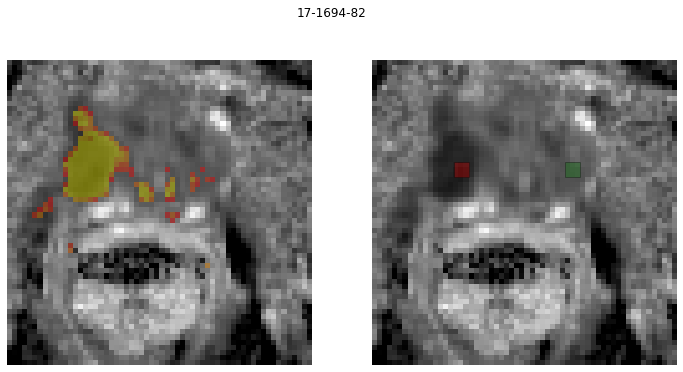


Figure S7: ERD Filtering for the ERC Patient 82.


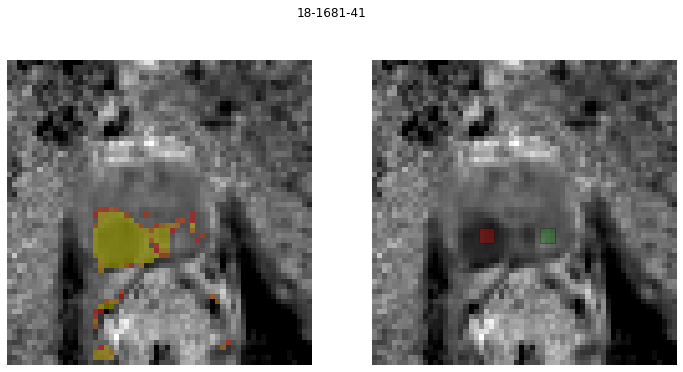


Figure S8: ERD Filtering for the ERC Patient 41.


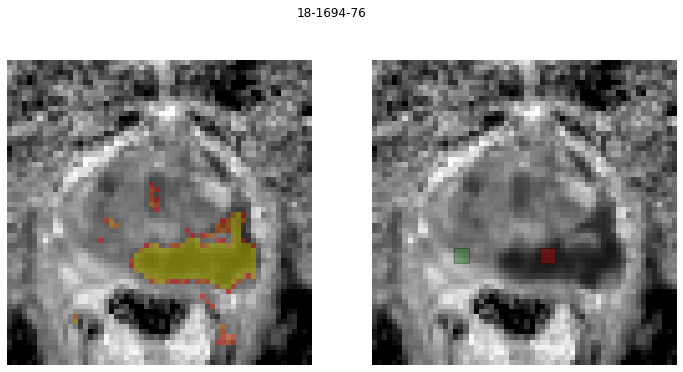


Figure S9: ERD Filtering for the ERC Patient 76.


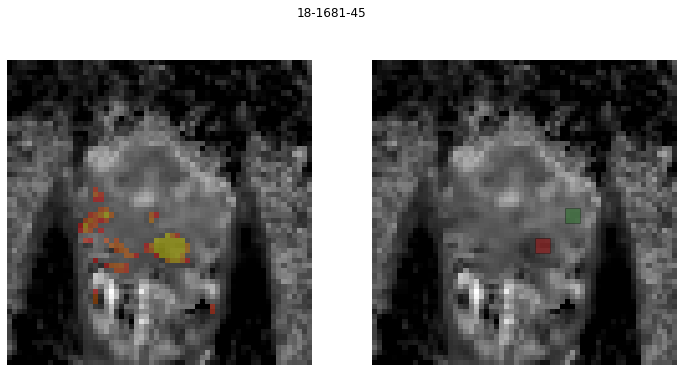


Figure S10: ERD Filtering for the ERC Patient 45.


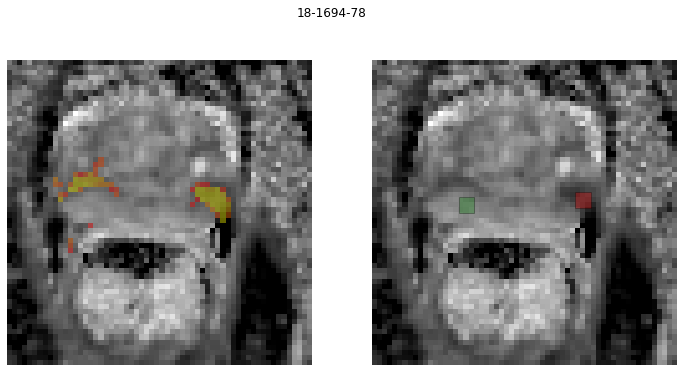


Figure S11: ERD Filtering for the ERC Patient 78.

1. **ERD WEIGHTING RESULTS FOR ALL PATIENTS**


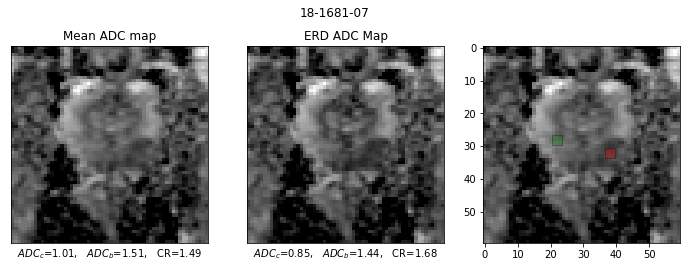


Figure S12: ERD Weighting for the NERC Patient 07. The enhancement due to the weighting can be observed on the middle image. The cancer and healthy areas are marked on the right image.


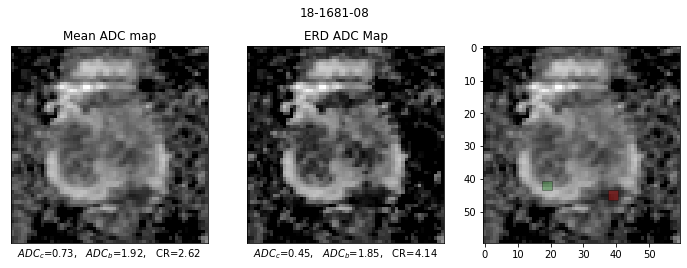


Figure S13 ERD Weighting for the NERC Patient 08. The enhancement due to the weighting can be observed on the middle image. The cancer and healthy areas are marked on the right image.


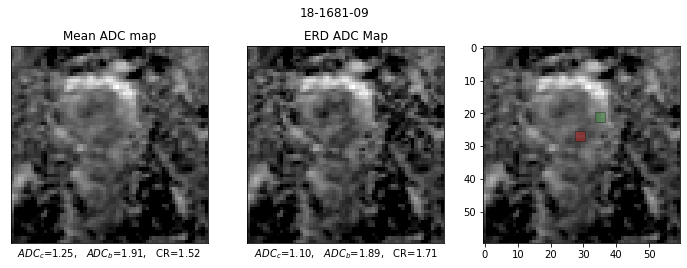


Figure S14 ERD Weighting for the NERC Patient 09. The enhancement due to the weighting can be observed on the middle image. The cancer and healthy areas are marked on the right image.


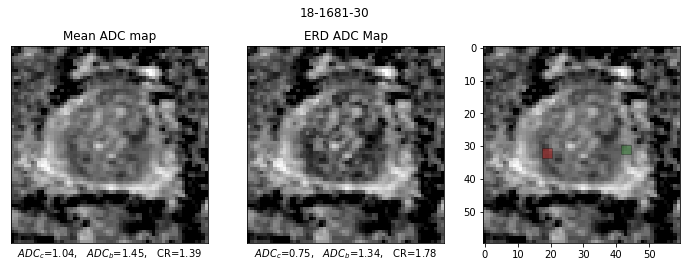


Figure S15 ERD Weighting for the NERC Patient 30. The enhancement due to the weighting can be observed on the middle image. The cancer and healthy areas are marked on the right image.


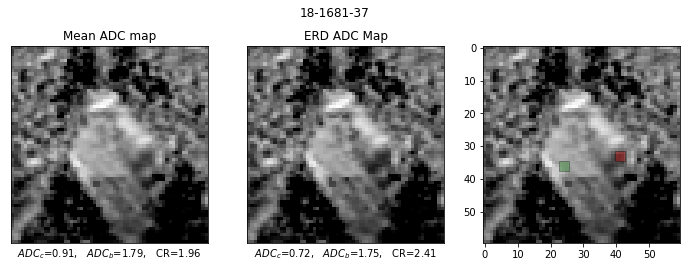


Figure S16 ERD Weighting for the NERC Patient 37. The enhancement due to the weighting can be observed on the middle image. The cancer and healthy areas are marked on the right image.


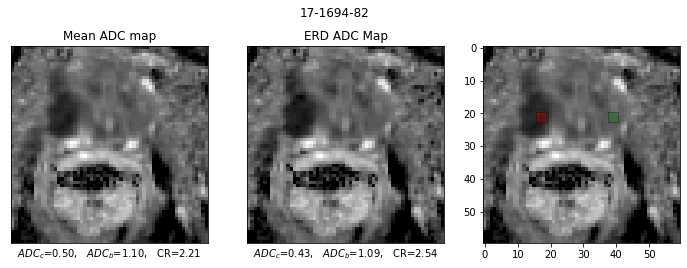


Figure S17 ERD Weighting for the ERC Patient 82. The enhancement due to the weighting can be observed on the middle image. The cancer and healthy areas are marked on the right image.


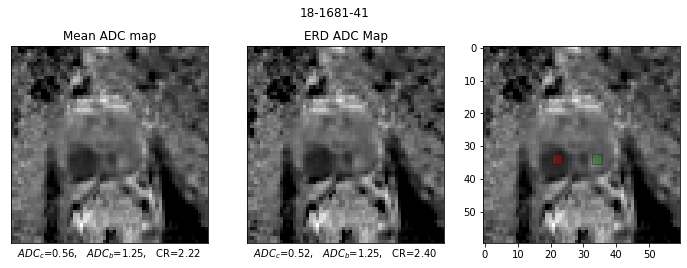


Figure S18 ERD Weighting for the ERC Patient 41. The enhancement due to the weighting can be observed on the middle image. The cancer and healthy areas are marked on the right image.


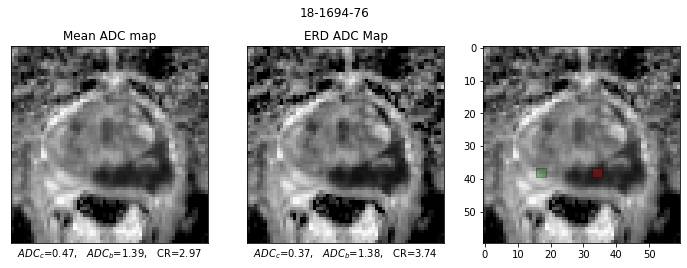


Figure S19 ERD Weighting for the ERC Patient 76. The enhancement due to the weighting can be observed on the middle image. The cancer and healthy areas are marked on the right image.


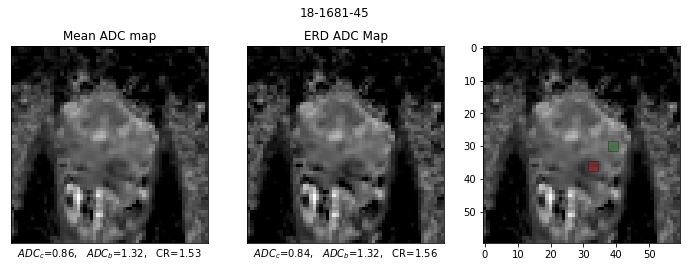


Figure S20 ERD Weighting for the ERC Patient 45. The enhancement due to the weighting can be observed on the middle image. The cancer and healthy areas are marked on the right image.


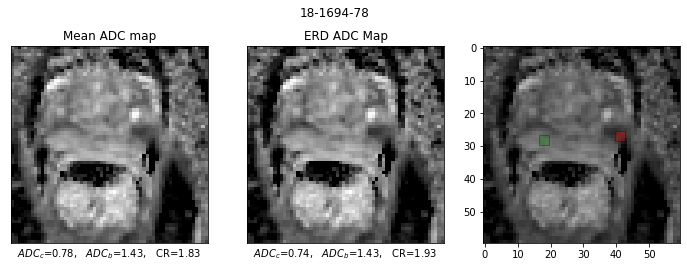


Figure S21 ERD Weighting for the ERC Patient 78. The enhancement due to the weighting can be observed on the middle image. The cancer and healthy areas are marked on the right image.


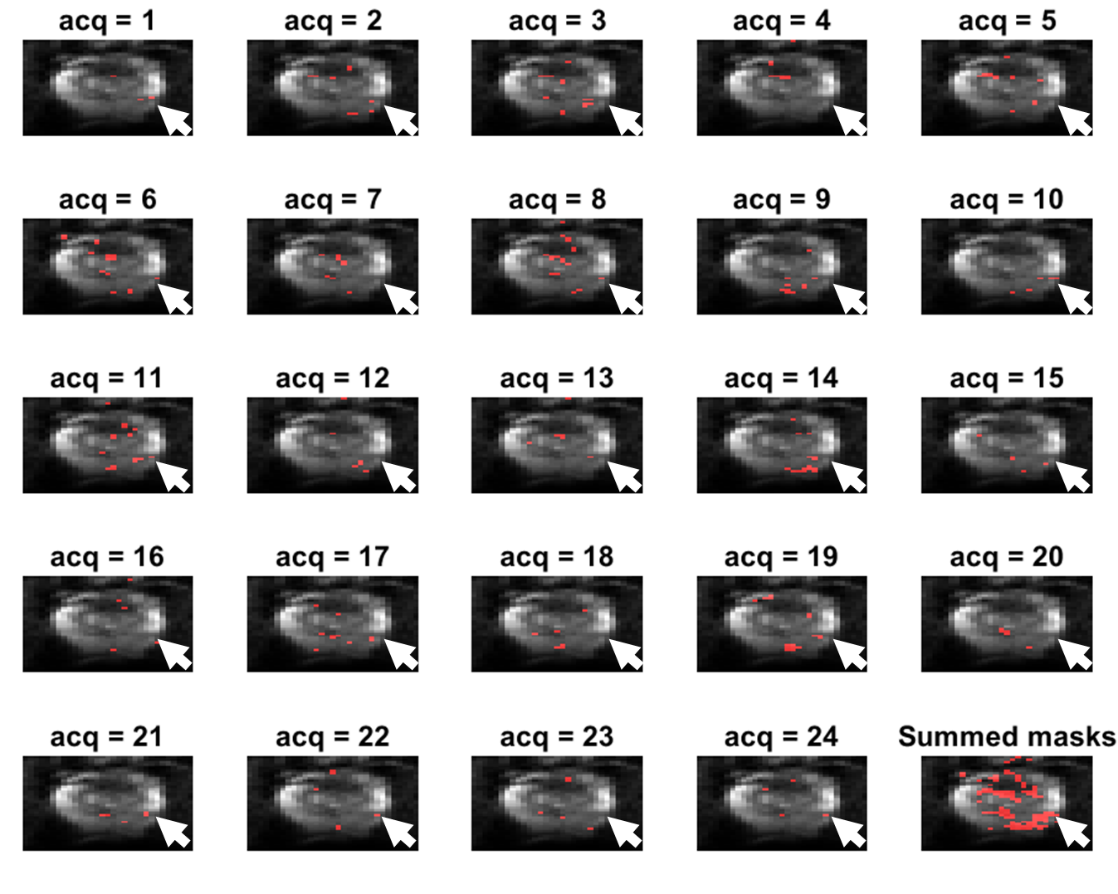


Figure S22: Visualization of inter-acquisition variability over all of the 24 independent acquisitions of a NERC patient, along with the map of the union of detections from all acquisitions. Red voxels show areas where the ADC is below the diagnostic
threshold of 1.04 × 10^−3^𝑚𝑚^2^∕𝑠 and the arrow shows the location of the biopsy-verified cancer.
